# Elderly Persons with Intellectual Disability: A Study of Clinical Characteristics, Functional Status, and Sensory Capacity

**DOI:** 10.1100/tsw.2003.24

**Published:** 2003-04-28

**Authors:** Eli Carmeli, Joav Merrick, Shlomo Kessel, Yossef Masharawi, Varda Carmeli

**Affiliations:** ^1^ Department of Physical Therapy, Sackler School of Medicine, Tel Aviv University, POBox 39040, IL-69978 Ramat Aviv, Israel; ^2^ National Institute of Child Health and Human Development, Division of Community Health, Zusman Child Development Center, Ben Gurion University, Beer-Sheva and Office of the Medical Director, Division for Mental Retardation, Ministry of Social Affairs, Jerusalem, Israel; ^3^ Neve Natoa Residential Care Center, Hadera, Israel; ^4^ Neve Ram Residential Care Center, Rechasim, Israel

**Keywords:** aging, functional ability, clinical characteristics, functional status, Israel

## Abstract

Longer life expectancy is resulting in increasing numbers of elderly adults with intellectual disability (ID). There has been the question whether persons with ID demonstrate early signs of aging before the general population. The aim of this study was to determine if persons with ID (with and without Down syndrome) showed premature aging changes compared with a control group. Elderly persons (n = 24, average age of 61) from one residential care center in Israel and younger adults from another center (n = 37, average age of 45) were compared with elderly residents without ID in an independent living facility. The study considered demographic data, medical data, anthropometric measurements, body fat and body mass index, flexibility, and sensorimotor function tests. The results showed that the persons with ID had basically similar body composition to that of persons without ID, however, the functional performance of elderly adults with ID was more impaired. We postulate that the slower functioning responses may be explained by a less physically active lifestyle, that may accelerate the onset of disease and result in symptoms associated with aging that are detrimental to health. It is therefore important that persons with ID participate in physical activity and exercises in order to promote health and prevent disease.

## INTRODUCTION

In recent years, aging has become an issue in the field of intellectual disability (ID), because longer life survival is resulting in increasing numbers of elderly adults with ID. This is also the case in Israel[1]. Researchers investigating aging processes have generally found similar aging patterns for people with or without ID[2]; nonetheless, individuals with ID show some characteristics of premature aging resulting in a greater inclination to morbidity and deconditioning[3,4,5]. Demographic aging, which refers to the increasing numbers of elderly adults within the overall population, is currently presenting major challenges for both health and social care services and for the immediate families.

Traditionally, intervention for aging persons with disabilities has focused on chronic physical impairment, functional capacity, and mental condition. Several approaches have been used to try and minimize secondary conditions and reduce mortality rates. Comparative studies of elderly individuals, with and without ID, have raised several questions and have also revealed significant differences between the two groups.

The prevalence of various diseases (diabetes, hypertension), obesity, decrease of flexibility, and sedentary living can be used as indications of premature aging[6]. Individual aging changes are very diverse and behavioral changes in day-to-day lifestyle have a major role in aging[7].

The prevalence of physically debilitating symptoms in aging persons with ID increases the demands for active diagnostic and therapeutic services. Such conditions include moderate to severe hearing loss and visual impairment[8,9], a high incidence of balance and gait dysfunction[10], arthritis and urinary incontinence[11], obesity[12], and nonischemic heart disorders[13]. Determination of premature aging may be indicated by a range of signs and symptoms[14]. The evaluation and active screening of cognitive and functional capabilities are important in order to assess their specific needs and to determine appropriate rehabilitative and psychotherapeutic programs.

The present study aims to evaluate and describe the physical and functional characteristics of elderly persons with ID in residential care and to compare their functional status and capabilities with a control population without ID, who lived in an independent living facility. This study tried to focus on functional capability rather than on impairment in adults with ID and we believe that its implications could influence the decision-making process in the welfare and health authorities.

## METHODS

### Population

A sample of elderly persons with intellectual disability (ID) was drawn from two residential care centers in Israel, Neve Ram in the north (total population 197 persons) and Neve Natoa in the central part (total population 166 persons). The first group consisted of 24 elderly adults (out of 34), hereafter referred to as **Group A1**, and included two subgroups: (*a*) with moderate ID and Down syndrome (DS) (n = 10) and (*b*) nonspecified moderate ID (NSID) (n = 14). The sample from the other center consisted of 37 (out of 46) younger adults, **Group A2**, and included two subgroups: (*a*) with moderate ID and DS (n = 13) and (*b*) NSID (n = 24). The participants gave written informed consent to the study through their legal guardians. All participants required minimal supervision for daily activities, and their daily activities regime was comparable.

The **control group (CG)** consisted of 31 (out of 42) older adults (aged 75.7 $\pm$ 5 years), who lived in independent living facilities located in Haifa in the north. All the participants of the CG provided informed consent to participate in the study. All participants with pre-existing conditions that could interfere with the results or could lead to comorbidity (with non-age–related changes) were excluded from the study (i.e., blindness, amputation, severe osteoarthritis). Comorbidity conditions included depression and possible adverse drug reactions.

Kappa statistics (K) were used to determine the inter-rater reliability for the different evaluation procedures. The evaluation procedures on separate days were undertaken by two physical and occupational therapists, with 4 and 19 years of clinical experience as geriatric therapists. All medical assessments were undertaken by the physicians of the residential care centers, who provided medical services to the facility for the last 7 years.

## Evaluation Procedures

The following evaluation procedures were included in the assessment protocol for each participant: demographic data, medical data, body composition, and two sensorimotor function tests.

Standard methods were used for anthropometric measurements including body weight (kg), height (cm), waist girth at the level of umbilicus, and thigh girth (at three points: the widest point, and 7 and 14 cm above the base of the patella) using a cloth measuring tape. To determine the waist:hip ratio (WHR), the waist measurement was divided by the hip measurement at the widest point. Healthy females and males should have WHR less than 1.8 and 1.9, respectively. Studies showed that females and males with ratios greater than 1.8 and 1.9 tended to have excessive fat around the waist. These measurements provide information about fat and muscle mass in the lower quadrant (i.e., pelvis and lower extremity) and serve as indicators for inclination to obesity, which are important because of their connection to health conditions such as heart disease and muscle strength[15].

Body fat determination (TBF 611, Tanita Corp. U.S.) is based on a bioelectrical impedance analysis method[16]. Bioelectrical impedance measures the length of time taken for an electrical impulse to travel from one place of the body (e.g., hand) to another place on the body (e.g., foot). The impulse requires less time to travel through muscle tissue than fat. Body mass index (BMI = weight[kg]/height[m2]) expresses the relationship of body weight to height. A BMI greater than 27.3 and 28.8 for females and males, respectively, is an indication of obesity[17].

Flexibility of the lower trunk and hamstring muscles was measured by procedures adapted from Hoeger[18] and the functional reach test of Duncan et al.[19] in a modified long-sitting and forward reach test. The test requires that the subject reach forward in a long-sitting position, while keeping the legs straight. The best score of three trials was recorded.

Two sensorimotor tests were performed: "timed-up and go" test (TUAG), and a 3-min distance walk test (3MDW). The TUAG test was used to measure the dynamic balance and gait speed[20]. A participant was asked to rise from an armchair, walk 3 m, and return to the chair. Times were measured using a manual stopwatch. The functional factors observed by the testers with this test include: path deviation, trunk sway, flexion of knees or back, abduction of arms, the use of a walking aid, sit-to-stand transfer, walk and turn. The target time period to complete this test for older adults with a good level of independence is between 8 and 10 s. The advantages of the test are that it is simple, requires simple tools, is quick to perform (less than 20 s including prior instructions), and can be performed by participants who use assistive devices such as a walker, cane, or crutches. The procedure was experimentally tested and found to be a highly reliable tool to measure balance function[21]. The 3MDW test was used to determine the number of steps (at self-selected walking speeds) and the distance the participant can walk in 3 min[21]. The test was performed in a quiet, obstacle-free outdoor area, 60 m long and 5 m wide. The participants walked between 10:00 and 11:00 a.m. in comfortable weather conditions (23ºC, 40% humidity). The monitor walked behind the participant so as not to influence the participant's pace, but as a safety precaution and to calculate the number of steps taken. Immediately after completing the walking test, the participant was seated and heart rate and blood pressure were evaluated.

## Statistical Analysis

All data were analyzed with SPSS 7.2 for Windows 97. Data are reported as mean ± one standard deviation. One sample independent *t*-test was run to compare differences in demographic and medical data, body composition, body fat, and sensorimotor tests between the groups. The critical value for statistical significance was assumed at an alpha level <0.05.

## RESULTS

Of the 28 elderly persons with ID initially selected for the evaluation from the first group, 24 (86%) were found eligible to participate in the study. Those dropped from the program had difficulties in complying with instructions or following specific orders.

Table 1 summarizes the demographic, body composition, and medical characteristics of all the participants. Group A1*a* (the aged ID group with DS) as expected, as well as group A1*b* (the NSID group), differed significantly from the CG (being younger with a higher percentage of females and widows or unmarried). Yet, groups A2*a,b* (the younger individuals with DS and with NSID) were more comparable to the CG in terms of gender. In comparison with the CG, groups A1*a,b* and A2*a,b* were fairly similar with regard to both body composition and medical problems with one exception, body weight was significantly lower ($p < 0.05$) from CG, demonstrated a higher incidence of cardiac disease ($p < 0.005$), and neurologic problems other than ID ($p <$ 0.001). Group A1*a* also showed significantly shorter heights and stature ($p < 0.05$). Individuals with DS showed more medical problems than individuals with NSID.

**TABLE 1**
**Demographic, Body Composition, and Medical Characteristics of Participants**

| | | Study Group<br>A1 DS<br>NSID | Study Group<br>A2 DS<br>NSID | CG | *p* Value |
|---|---|---|---|---|---|
| Participants (number) | | 10<br>14 | 13<br>24 | 31 | |
| Age (year) | | 59 ± 4<br>61 ± 3 | 44 ± 3<br>45 ± 4 | 75 ± 7 | <0.001 |
| Gender (%) | M/F | 9/91<br>11/89 | 48/52<br>52/48 | 16/84 | NS |
| Body weight (kg) | M | 66.6 ± 4.4 | 70.4 ± 5 | 65.2 ± 4.9 | <0.05 |
| | F | 67.1 ± 3.6 | 69.8 ± 7 | 65.3 ± 4.4 | NS |
| | M | 64.1 ± 4 | 68.4 ± 6 | | <0.05 |
| | F | 63.4 ± 4.3 | 67.6 ± 6 | | <0.05 |
| Height (cm) | | 149.4 ± 5.6<br>152 ± 4.9 | 158.7 ± 6.2<br>160 ± 5.5 | 160.7 ± 3.3 | <0.05 |
| Body fat (%) | | 21<br>20 | 21<br>19 | 20 | NS |

**TABLE 1 (continued)**

| | | Study Group A1 DS NSID | Study Group A2 DS NSID | CG | *p* Value |
|---|---|---|---|---|---|
| Waist girth (cm) | M | 87.2 ± 3.2 | 86.5 ± 2.2 | 84.3 ± 3.3 | NS |
| | F | 89.3 ± 3.4 | 89.9 ± 8.8 | 87.5 ± 4.4 | |
| | M | 85.2 ± 2.5 | 87.7 ± 7.2 | | |
| | F | 86.6 ± 3.1 | 86.4 ± 4.5 | | |
| Thigh girth (cm) | M | 50.5 ± 3.2 | 51.7 ± 4.7 | 49.4 ± 3.7 | NS |
| widest point (M/F) | F | 49.0 ± 4.1 | 50.9 ± 5.7 | 52.2 ± 4.9 | |
| | M | 58.9 ± 3.6 | 50.5 ± 5.1 | | |
| | F | 51.3 ± 4.4 | 51.7 ± 4.9 | | |
| WHR | M/F | 1.7<br>1.8 | 1.7<br>1.7 | 1.7 | NS |
| BMI | M | 26.1 | 25.9 | 25.2 | NS |
| | F | 26.8 | 25.6 | 25.9 | |
| | M | 26.2 | 25.4 | | |
| | F | 26.3 | 25.7 | | |
| Single/widowed (%) | | 100<br>100 | 95<br>100 | 78 | <0.001 |
| Cardiac disease* (%) | | 39.7<br>11.9 | 30.4<br>2.3 | 26.4 | <0.005<br><0.05 |
| Hypertension (%) | | 38.8<br>21.3 | 28.2<br>3.3 | 31.8 | NS<br><0.05 |
| Diabetes (%) | | 17.6<br>2.6 | 10.3<br>0 | 19.9 | <0.005<br><0.05 |
| Respiratory disease (COPD, asthma) (%) | | 27.3<br>20.1 | 25.5<br>13.5 | 24.5 | <0.001<br><0.05 |
| Hepatic disease (%) | | 10.6<br>0 | 5.0<br>0 | 11.1 | <0.005<br><0.05 |
| Neoplastic disease (benign and malignant tumors) (%) | | 3.6<br>0 | 0<br>0 | 4.1 | <0.005<br><0.05 |
| Renal disease (insufficiency and chronic UTI) (%) | | 9.6<br>0 | 7.3<br>0 | 10.6 | <0.005<br><0.05 |
| Neurologic disease (%) | | 40.7<br>45.8 | 37.6<br>51.2 | 11.5 | <0.001 |
| Vascular disease (%) | | 19.6<br>0 | 9.9<br>0 | 11.9 | <0.005<br><0.05 |
| GI diseases (%) (gastritis, duodenal ulcer, chronic constipation) | | 15.8<br>20.2 | 11.7<br>5.6 | 18.9 | <0.005 |
| Medication (no.) | | 3<br>4 | 4<br>2 | 4 | NS |

DS: Down syndrome; NSID: nonspecified ID; NS: nonsignificant; M: male; F: female; $p < 0.05$.

*Cardiac diseases include ischemic heart or congestive heart failure.

**TABLE 2**
**Sensorimotor Tests**

| | A1 Group (n = 24)<br>A2 Group (n = 37) | | | CG (n = 31) | | | |
|---|---|---|---|---|---|---|---|
| | **Male<br>(n = 4)<br>n = 16** | **Female<br>(n = 20)<br>n = 21** | **Mean** | **Male<br>(n = 10)** | **Female<br>(n = 21)** | **Mean** | **Difference<br>%** |
| Flexibility (cm) | 20<br>21.4 | 18<br>22 | 18.7<br>21.2 | 21 | 19.4 | 20.3 | **7.8 |
| TUAG (s) | 12.9<br>7.2 | 13.3<br>6.9 | 13.2<br>7.0 | 7.4 | 7.2 | 7.3 | **80.8 |
| 3MDW (m/steps) | 131/274<br>165/349 | 128/270<br>160/342 | 129/272<br>162/340 | 152/309 | 149/307 | 150/308 | **14 |

CG: Control group; A1: elderly ID; A2: younger ID.
** $p < 0.05$ between A1 and CG.

Table 2 shows the results of the flexibility and the two sensorimotor tests. Functional flexibility tests showed only slight and insignificant differences between the three groups. The ID study groups (A1 and A2 with DS and NSID) and the CG were able to bend forward in long-sitting positions for 18.7, 21.2, 20.3, and 22.6 cm, respectively. Group A1 demonstrated significantly poorer performance in both sensorimotor tests than group A2 and the CG ($p < 0.05$). Times longer than 12 s to perform the TUAG test may indicate some balance difficulties and increased risk for falling. In both the 3MDW test and the walking velocity test, group A1*a,b* walked significantly shorter distances at a slower pace (mean of 129 m in 272 steps, average of 90 steps per minute) than A2*a,b* (mean of 162 m in 340 steps, average of 116 steps per minute); CG (mean of 150 m in 308 steps, average of 102 steps per minute), $p < 0.05$.

Table 3 shows the inter-rater reliability results for specific evaluation items. The Kappa statistic was used to determine the amount of agreement between the therapists' assessment, thereby establishing the inter-rater reliability between them. The Kappa statistic was chosen, because it is applicable to categorical variables and because it assesses agreement beyond what may be expected based on chance alone. According to Landis and Koch[22], Kappa values greater than 0.75 represent excellent agreement between raters. The entire evaluation procedure took an average of 41 min to administer. The Kappa values for participant evaluations ranged from 0.66 to 0.94 as indicated in Table 3. All items except medical history taken (0.69) were in excellent agreement.

## DISCUSSION

The main purpose of this study was to compare the clinical and functional characteristics between populations of aging adults with and without ID. The study examined longitudinallly the test scores gained by different age cohort of older and younger adults with DS and with NSID. As well, the study examined cross-sectionally the test scores gained by different age cohorts of adults without ID. Standard evaluations were used for elderly adults and younger with ID in residential care as compared with CG residing in independent care facilities, and the mean age of the CG was 14 years older than that of the group A1. We were unable to use age-matched samples owing

**TABLE 3**
**Inter-Rater Reliability Results for Evaluation Items for the Group of Older Persons with ID (A1, DS, and NSID) and CG**

| Evaluation Item | Study group (A1) (n = 24) | | CG (n = 31) | | Kappa Study/CG |
|---|---|---|---|---|---|
| | Agree | Disagree | Agree | Disagree | Study/CG |
| Weight | 23 | 1 | 31 | 0 | 0.94/1.0 |
| Height | 23 | 1 | 30 | 1 | 0.94/0.94 |
| Body fat | 24 | 0 | 31 | 0 | 1.0/1.0 |
| Thigh and waist girth | 22 | 2 | 29 | 2 | 0.79/0.80 |
| Flexibility | 23 | 1 | 29 | 2 | 0.79/0.80 |
| Sensorimotor tests | 22 | 2 | 30 | 1 | 0.79/0.89 |
| Medical history taken | 21 | 3 | 28 | 3 | 0.66/0.69 |

to the limited numbers of individuals with ID older than 63 years of age living in Israel. Moreover, the effects of long-term residential care and the manifestation of institutionalization were important in this study, but the number of non-ID institutionalized individuals was extremly limited and therefore there the match was not aged matched in this study, but rather accomodation matched.

It is commonly believed that older adults with ID tend to demonstrate premature signs of aging, characterized by changes in body composition, functional decline, and increased morbidity. How early in their life they present these signs is yet to be investigated, however in our study we could see that premature aging was not presenting in the younger group (45 years old). The relatively few previous aging investigations concerning elderly adults with ID have suggested that persons with ID develop some aging characteristics earlier than non-ID adults[23,24]. The present study provides added support for the viewpoint of premature aging in persons with ID. Burt et al.[25] examined the changes in functioning with aging in a longitudinal study in adults (22 to 56 years old) with DS and concluded that there is only minimal age-related declines in functioning. Their study did not support the idea that adults with DS show rapid age-related declines before age of 50. Our findings demonstrate that young individuals of 45 years old do not show earlier signs of aging.

The results of our present study indicate that body composition of adults with ID (61 years old), including weight, height, BMI, WHR, and body fat, are similar to those in our CG (75 years old). Changes in body composition represent one of the more dramatic biological markers of advanced age and are especially pronounced after the age of 70 years[26]. Although DS individuals are generally of short stature, their height differences compared to the control group were insignificant. Although the mean age of the control group was 14 years greater than that of the A1 group, the study showed no significant differences between the two groups with regard to body composition. It seems to indicate that accelerating aging seen in the ID group might be indirectly reflected by body composition values. Age-associated loss of muscle mass, sarcopenia[27], with concomitant loss of strength and decreased joint flexibility and mobility, contribute to frailty in the older adults and expose these individuals to increased risk[28,29,30,31,32].

Our results lead us to assume that relative inactivity in adults may also lead to premature aging changes in body composition and may constitute increased risk for losing independence, and that the A1 group appears equally at risk to that of the older control group.

Most of the medical histories revealed nonsignificant differences between the A1 group and CG with two exceptions. Heart disorders were considerably more prevalent among individuals with ID. These heart disorders were often congenital, diagnosed early, and well treated. The incidence of neurological diseases in the A1 group (as demonstrated in Table 1) supported the notion that these individuals will probably require more assistance and care in the future. An early onset of medical problems and prolonged poor health might accelerate the aging processes. It is noticiable that DS individuals possed more medical problems than individuals with NSID with only one execption of neurological diseases. This may reflect a predisposition to health problems among individuals with DS.

Functional flexibility of the trunk flexion and hamstring muscle stretching were similar in both groups. This indicated that both study groups and CG demonstrated similar degrees of soft tissue mobility and lumbar flexibility despite their differences in age. Loss of flexibility is one of the earliest noticeable changes occurring in elderly adults aged 70 years or older[33]. The fact that 61-year-old individuals with ID demonstrate similar degrees of flexibility to that of 75-year-old non-ID individuals may possibly be a consequence of a more passive lifestyle. It may be associated with the constant help and assistance they receive in their daily living activities and may reflect a "use it or lose it" phenomenon. The flexibility results are somewhat surprising and merit special attention. Since individuals with DS often possess poor skeletal muscle tone with soft tissue elasticity, one might have expected a relative disadvantage with respect to flexibility as they aged, however, this was not the case. Since we did not evaluate muscle tone in the present study, we can only assume that a "lazy" lifestyle probably played a major role in determining flexibility levels at age 60 among DS individuals compared to those of 75-year-old individuals without ID.

The TUAG test is one of the more user-friendly functional assessment tests in the clinic, whereas the 3MDW test is an outdoor functional test used to assess the endurance capacity of the skeletal muscle of lower extremities, the heart, and lungs[34]. Group A1 scored lower in the functional tests, which may reflect on their general state of health or sedentary lifestyle and may indicate an increased mild risk for falling. The TUAG test was used to assess a person’s ability to perform a task that is probably performed many times throughout the day by elderly adults. Adults, who take more than 20 s to complete the test are at high risk of falling and are not considered safe to be alone outside. This test can also supply further information about the participant’s functional ability and rehabilitation requirements. Poor performance (slower than 20 s) may be used to justify therapy intervention as a preventive measure. Group A1 needed significantly more time to complete the test than the control group, but still finished the test within the normal timeframe. On-going follow-up is needed to detect, sooner rather than later, any deterioration in performing that specific task.

In the 3MDW test at self-selected walking speeds, the A1 group covered shorter distances and were significantly slower than the A2 group and the CG. According to Bohannon et al.[35] a paced walking speed for elderly adults of 70 steps per minute is considered a slow walking velocity, and paced walking speed of 80 steps per minute is considered a slow-to-moderate walking speed. One explanation for our data may be that daily living and recreational activities of persons with ID in residential care do not provide sufficient stimuli to preserve functional mobility to the extent found in the CG. Consequently, results indicating low endurance capacity may conceivably reflect either poorer health conditions or less-intense life activities and a tendency for deconditioning[36,37].

Several improvements in the evaluation procedures may warrant consideration. For example, use of magnetic resonance imaging (MRI) as a reference method for quantifying the muscle mass is more accurate, but significantly more expensive and time-consuming[38].

## CONCLUSIONS

Our findings found that aged individuals with ID do present several indications of premature aging and in particular those with DS. The mechanisms, although still unknown, may be associated with physically inactive lifestyles, which in DS individuals may lead to acceleration of functional disabilities and overall less favorable health as they age. Further information concerning psychological and behavioral characteristics of inactive older adults will be useful for funneling diagnostic and preventive measures to the most vulnerable groups such as the individuals with ID.

## BIOSKETCH

**Eli Carmeli,** P.T., Ph.D., is currently working in a full-time tenure tract position at the Physical Therapy Department, Sackler Faculty of Medicine, Tel Aviv University. He is investigating the aging process both on the cellular and clinical level.
